# Efficacy and Safety of Laser Therapy and Phototherapy in Cicatricial and NonCicatricial Alopecia: A Systematic Review Study

**DOI:** 10.1002/hsr2.70180

**Published:** 2024-11-04

**Authors:** Mohammad Amin Jafari, Ghazal Bazgir, Fatemeh Sadat Hosseini‐Baharanchi, Alireza Jafarzadeh, Azadeh Goodarzi

**Affiliations:** ^1^ Department of Dermatology, Rasool Akram Medical Complex Clinical Research Development Center (RCRDC), School of Medicine Iran University of Medical Sciences (IUMS) Tehran Iran; ^2^ Student Research Committee, School of Medicine Iran University of Medical Sciences Tehran Iran; ^3^ Department of Biostatistics, School of Public Health Iran University of Medical Sciences Tehran Iran

**Keywords:** alopecia, alopecia noncicatrisata, baldness, cicatrisata, low‐level light therapy, lupus erythematosus

## Abstract

**Background and Aims:**

In recent years, the application of various light and laser devices in the treatment of different types of alopecia has been established. This systematic review aims to assess the efficacy and safety of laser therapy and phototherapy in cicatricial and non‐cicatricial alopecia.

**Methods:**

A comprehensive search was conducted on PubMed, Scopus, Science Direct, and Google Scholar. Articles were evaluated across four subgroups: alopecia areata, androgenic alopecia, telogen effluvium, and cicatricial alopecia. Included studies were published in English or Persian between January 2010 and September 2023, focusing on interventional, cohort, or case series research that achieved a minimum score of 75% on the EBL checklist. Exclusion criteria encompassed animal and in vitro studies, review articles, case reports, duplicated or irrelevant research, as well as studies that did not meet the designated EBL score. Editorial letters and case studies were also excluded.

**Results:**

Initially, 965 records were collected, resulting in the inclusion of 58 studies in the final review: 26 on alopecia areata, 26 on androgenic alopecia, five on cicatricial alopecia, and one on telogen effluvium. Narrow‐band ultraviolet B, 308‐nm excimer laser, and psoralen ultraviolet A therapy showed varying effectiveness; specifically, the excimer laser was notably effective for patients with shorter disease duration. In androgenic alopecia, erbium‐glass and thulium lasers effectively increased hair density but showed a gradual decline posttreatment. Low‐level light/laser therapy also increased hair density and diameter and exhibited potential benefits when used alongside minoxidil, but did not significantly enhance outcomes in telogen effluvium treatment.

**Conclusion:**

Light/laser therapy can serve as an additive treatment for cicatricial alopecia, particularly lichen planopilaris, but has limited efficacy in treating telogen effluvium. Overall, light/laser therapies exhibit a significant positive effect on increasing hair density and diameter across various alopecia types.

**Abbreviations**
AA: alopecia areata
AGA: androgenic alopecia
DLE: discoid lupus erythematous
FFA: frontal fibrosing alopecia
FPHL: female pattern hair loss
LLLT: low‐level light/laser therapy
LPP: lichen planopilaris
MPHL: male pattern hair loss
PUVA: psoralen ultraviolet A
RCT: randomized control trial
TE: telogen effluvium
UVA: ultraviolet A
UVB: ultraviolet B

## 1 Introduction

Alopecia is a common skin disorder that affects the population all over the world. This skin disorder can affect people's mental health as well as their quality of life [1]. In other words, this disorder can increase psychological disorders such as depression, anxiety, social phobia, posttraumatic stress disorder, and suicidal ideas and this is more severe with cicatricial types of alopecia [2]. Approximately 50% of men and women experience alopecia during their lifetime [3].

Alopecia is categorized into cicatricial (scarring) and noncicatricial (nonscarring) types [4]. Major subgroups of noncicatricial alopecia include androgenic alopecia (male or female pattern), alopecia areata, hair cycle disorders (telogen or anagen effluvium), Lichen planopilaris, frontal fibrosing alopecia and discoid lupus erythematosus [5]. In noncicatricial alopecia, the stem cells of the hair follicle located in the area of the bulge retain their regrowth potential. While in the cicatricial type, these cells are destroyed, which leads to permanent alopecia [6, 7, 8].

Androgenetic alopecia (AGA) is the most common cause of noncicatricial alopecia, which increases with age after puberty. Androgenetic alopecia affects 50% of women and 80% of men [9, 10]. Its prevalence is higher in Caucasians than in African‐Americans and Asians [11, 12]. Many types of alopecia have a chronic nature or can lead to permanent complications. Therefore, they need a long period of treatment and control. Various treatment choices for alopecia are available from medical to more aggressive surgical ones for example platelet‐rich plasma (PRP) [13] and hair transplantation, but it can be said that for many alopecia types except types with FDA‐approved treatments, an exclusive treatment method is not available and responses of patients to the same treatment may be different. In the past few years, the use of autologous PRP has been a popular method in the treatment of hair loss, as it increases hair survival and growth. However, few studies have been published on the effectiveness of this method in the treatment of Alopecia [14]. Alopecia treatment with the laser has recently been considered by researchers, especially in male and female pattern hair loss, but its use is far less than other methods [15]. The epidermal stem cells found in the follicular ridge are stimulated by the laser, thus causing the follicular to change from the anagen phase to the telogen phase. In addition, it increases the duration of anagen and causes vasodilation and increased blood flow [16]. The used methods in the treatment of alopecia must be available, cost‐effective, and have the least adverse effects. Some studies have reported, laser and phototherapy in treating Alopecia with positive outcomes, with fewer side effects; others revealed the opposite. Given this inconclusive issue, it is essential to integrate and compare these findings in the secondary analysis. So the current study aimed to assess the efficacy and safety of laser therapy and phototherapy in cicatricial and noncicatricial alopecia in a systematic review.

## 2 Materials and Methods

### 2.1 Study Design

**Figure 1:**
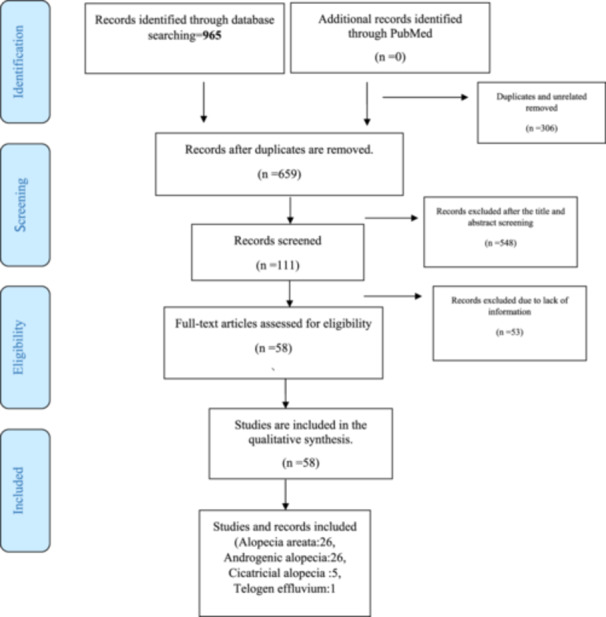
PRISMA flow diagram for included studies in the current meta‐analysis.

This study was implemented according to the PRISMA (Preferred Reporting Items for Systematic Reviews and Meta‐Analyses) statement, adhering to CONSORT guidelines to ensure clear reporting standards. A PRISMA flow chart illustrating the study selection process is presented in Figure 1. Statistical analyses followed guidelines from SAMPL (Statistical Analyses and Methods in the Published Literature).

### 2.2 Ethical Approval

The study received ethical approval from the Iran University of Medical Sciences, with the ethics code number IR.IUMS.FMD.REC.1401.127, to ensure compliance with ethical standards. Informed consent was obtained from all participants before their inclusion in the study.

### 2.3 Study Selection

Inclusion criteria encompassed studies published in English or Persian from January 2010 to September 2023, focusing on the efficacy of laser therapy and phototherapy for both cicatricial and noncicatricial alopecia. Eligible study types were interventional, cohort, and case series, with a required minimum score of 75% on the EBL checklist to ensure study accuracy.

The PICO framework includes:

Population: Adult patients of any gender diagnosed with cicatricial or noncicatricial alopecia.

Intervention: Laser therapy and phototherapy.

Comparison Outcome: Evaluated based on the investigator's global assessment (IGA) of hair regrowth, treatment satisfaction, hair count, thickness, density (measured in number per cm²), phototrichogram results, or SALT score solely for alopecia areata, as well as the identification of any side effects (e.g., eczema, pruritus, acne, erythema, infiltration, hyperkeratosis, stinging sensation, skin dryness, pain, or ulceration).

Exclusion criteria included animal studies, in vitro studies, case reports, review articles, duplicated or irrelevant studies, and those that did not meet the EBL checklist score. Editorial letters, case studies, and articles lacking sufficient data were also excluded.

### 2.4 Search Strategy

This systematic review utilized international electronic databases, including PubMed, Scopus, Science Direct, and Google Scholar. The search incorporated keywords such as Alopecia, Baldness, Hair Loss, Androgenetic Alopecia, Alopecia Cicatrisata, Alopecia Areata, and Phototherapy (refer to Table 1). The Endnote® 20 software (Clarivate Analytics, Philadelphia, USA) facilitated study screening and data extraction. Two researchers independently executed the search strategy, screening titles and abstracts initially, followed by independent review of full texts. Articles were assessed based on methodology, sampling methods, reliability of assessment tools, and study objectives. Discrepancies in the inclusion process were resolved through discussion without the need for third‐party involvement.

**Table 1:** Search syntax across databases.

PubMed	(“Alopecia”[Title/Abstract] OR “Baldness”[Title/Abstract] OR “Hair loss”[Title/Abstract] OR “Androgenetic Alopecia”[Title/Abstract] OR “Androgenic Alopecia”[Title/Abstract] OR “Alopecia Cicatrisata”[Title/Abstract] OR “Alopecia Areata”[Title/Abstract] OR “Telogen Effluvium”[Title/Abstract] OR “Discoid lupus erythematosus”[Title/Abstract] OR “Lichen Planopilaris”[Title/Abstract] OR “Frontal fibrosing alopecia”[Title/Abstract]) AND (“Phototherapy” “[Title/Abstract] OR “Laser Therapy” [Title/Abstract] OR “Lasers”[Title/Abstract] OR “Excimer”[Title/Abstract] OR “YAG Laser” [Title/Abstract] OR “Low‐Level Light Therapy”[Title/Abstract] OR “LLLT”[Title/Abstract] OR “Ultraviolet Therapy”[Title/Abstract] OR “Photobiomodulation”[Title/Abstract] OR “PUVA Therapy”[Title/Abstract] OR “Psoralen Ultraviolet A Therapy”[Title/Abstract] OR “narrow band UVB” [Title/Abstract] OR “UVA” [Title/Abstract] OR “UVB”[Title/Abstract] OR “UV”[Title/Abstract]) AND (2010:2023[pdat])
Scopus	(TITLE‐ABS‐KEY (“Alopecia” OR “Baldness” OR “Hair loss” OR “Androgenetic Alopecia” OR “Androgenic Alopecia” OR “Alopecia Cicatrisata” OR “Alopecia Areata” OR “Telogen Effluvium” OR “Discoid lupus erythematosus” OR “Lichen Planopilaris” OR “Frontal fibrosing alopecia”)) AND TITLE‐ABS‐KEY (“Phototherapy” OR “Laser Therapy” OR “Lasers” OR “Excimer” OR “YAG Laser” OR “Low‐Level Light Therapy” OR “LLLT” OR “Ultraviolet Therapy” OR “Photobiomodulation” OR “PUVA Therapy” OR “Psoralen Ultraviolet A Therapy” OR “narrow band UVB” OR “UVA” OR “UVB” OR “UV”) AND PUBYEAR > 2009 AND PUBYEAR < 2025
Web of Science	TS = (“Alopecia” OR “Baldness” OR “Hair loss” OR “Androgenetic Alopecia” OR “Androgenic Alopecia” OR “Alopecia Cicatrisata” OR “Alopecia Areata” OR “Telogen Effluvium” OR “Discoid lupus erythematosus” OR “Lichen Planopilaris” OR “Frontal fibrosing alopecia”) AND TS = (“Phototherapy” OR “Laser Therapy” OR “Lasers” OR “Excimer” OR “YAG Laser” OR “Low‐Level Light Therapy” OR “LLLT” OR “Ultraviolet Therapy” OR “Psoralen Ultraviolet A Therapy” OR “narrow band UVB” OR “UVA” OR “UVB” OR “UV”)

### 2.5 Risk of Bias Assessment

To assess risk of bias in clinical trials, the Jadad scale was employed, which a 5‐point scale is evaluating sequence generation, blinding, and withdrawals. A score above 3 is indicative of high quality [17]. For cohort studies, the Newcastle–Ottawa Scale was utilized, which considers:

Selection of Participants: Four items assessing the representativeness of the cohort, derivation of exposed/nonexposed participants, ascertainment of exposure, and the absence of outcome at the study's initiation.

Comparability: Evaluated through one item analyzing the comparability of cohorts based on study design.

Outcomes: Assessed via three items regarding outcome assessment, follow‐up time, and adequacy of follow‐up. The maximum score is 9, with scores of 0–3, 4–6, and 7–9 corresponding to low, moderate, and high‐quality studies, respectively [18]. For case series studies, a risk of bias assessment was not performed.

### 2.6 Statistical Analysis

Statistical analyses were performed using [SPECIFY STATISTICAL SOFTWARE AND VERSION], with a significance level set at [SPECIFY *α*, e.g., *p* < 0.05]. Both one‐sided and two‐sided tests were employed, as appropriate for the analyses. Pre‐specified analyses included [DESCRIBE PRE‐SPECIFIED ANALYSES], while exploratory analyses focused on [DESCRIBE EXPLORATORY ANALYSES OR SUBGROUP ANALYSES]. Statistical terms and abbreviations were defined as follows: [DEFINE TERMS/ABBREVIATIONS].

## 3 Results

### 3.1 Study Selection

A total of 965 records were retrieved through electronic databases, with 306 duplicate records deleted. Authors screened 659 articles. Of these, 548 articles were excluded after title and abstract screening in the first step, and an additional 53 articles did not meet the inclusion criteria in the second step due to a lack of information. Thus, 58 records were included in the final review: 26 studies on alopecia areata (Table 2), 26 on androgenic alopecia (Table 3), five on cicatricial alopecia (Table 4), and one on telogen effluvium (Table 5).

**Table 2:** Summary of results in Alopecia areata.

	First author, year	Study type	Light/laser type	Sample size	Sex	Intervention	Laser/light features	Judgment criteria	Results	Side effect	Risk of bias
1	Sandeep Kaur (2015) [19].	Clinical trial	NBUVB	40 Patients (120 patches)	28 M, 12 F	6 weeks, twice a week, patch1 with intralesional triamcinolone, patch2 with NBUVB, patch3 both	initial dose of 300 mJ/cm^2^ with increments of 10% at every visit, till MED (minimal erythema dose)	G0 no growth, G1 < 25% regrowth (poor), G2 < 50% regrowth (fair), G3 < 75% regrowth (good), G4 complete regrowth (excellent)	P1: more than 50% response in 67.5% (with 35% G4) P2: more than 50% response in 17.5% (with 12.5% G4) P3: more than 50% response in 62.5% (with 32.5% G4)	Transient itching or redness in the treated area	3
2	Moustafa A. El Taieb (2019) [20].	Clinical trial	NBUVB	60 Patients	28 M,32 F	3 months, twice a week, group1 topical calcipotriol, group2 NBUVB, group3 both, group4 placebo	311 nm by eight narrowband UVB lamps (TL01) of Waldman‐type F 85/100W‐01. initial dose of 0.25 J/cm^2^ with increments of 10%–20% at every visit, with a maximum dose of 740 mJ/cm^2^ according to MED	SALT score and vitD3 levels	Group2: Salt score: before (3.97 ± 0.7), after (2.33 ± 0.7), *p*‐value 0.005 serum vit D level: before (33.37 ± 4.1), after (40.77 ± 4.7), *p*‐value 0.011	Not reported	4
3	DilekBayramgürler (2011) [21].	Cohort	NBUVB	25 Patients	9 M, 16 F	30–113 sessions, thrice a week, then after terminal hair regrowth, then once per week	A Cosmedico cabinet, the initial dose of 0.2 or 0.3 J/cm^2^ with increments of 20% at every visit to max. 1.8 J/cm^2^	SALT score	Before: S3: 9, S4: 6, S5: 3, S5B: 7 after: S1: 12, S2: 4, S3: 3, S4: 6, *n* = 25	Not reported	5
4	Kübra ESEN SALMAN (2019) [22].	Cohort	NBUVB	34 Patients	21 M, 13 F	23.76 ± 13.60 (2–54) sessions, twice to thrice a week,	A Daavlin–Levia device, Initial dose at 50% of MED with increments of usually 20% every session	No response (0%–25%), mild response (25%–50%), moderate response (51%–75%), significant response (76%–90%), and complete response ( > 90%)	No response 9, mild 10, moderate 4, significant 0, complete 9, n = 32 An excellent response was obtained in 52.9% of Patients with alopecia areata.	Mild to severe erythema	7
5	Ji Won Byun (2015) [23].	Clinical trial	308‐nm excimer laser therapy	8 Patients	3 M,5 F	12 weeks, twice a week, alopecic patch divided into control and treated side	XTRAC®, Initial at a dose of 50 mJ lower than the MED with increments of 50 mJ every week	Hair diameter and hair count (vellus and terminal)	Hair diameter: before (treated 0.0435 ± 0.014) and (control 0.0429 ± 0.022 mm), after (treated 0.0681 ± 0.018) and (control 0.043 ± 0.009 mm), *p* = 0.012 in treated side, *p* = 0.013 compared to control Hair count: before (69.38 ± 46.07/cm^2^), after (122.63 ± 58.96/cm^2^), *p* = 0.018 Terminal hair count: before (60.38 ± 55.17), after (88.38 ± 82.47), Vellus hair count: before (9 ± 12.66), after (87.25 ± 72), *p* = 0.043	Mild pain, erythema	4
6	Zo Nun Sanga (2015) [24].	Clinical trial	308 nm excimer light	30 Patients	23 M, 7 F	8 Weeks or till total growth, twice a week, alopecic patch divided in control and treated side	A Xenon chloride lamp, the initial dose of 300 mJ/cm^2^, with increments of 100 mJ/cm^2^ at every sitting until MED	< 25% hair regrowth (poor response – Grade 1), 25–49% (fair response – Grade 2), 50–74% hair regrowth (good response – Grade 3), and 75%–100% hair regrowth (excellent response – Grade 4)	G1: [treated 18 (60%), control 29 (96.67%)], G2: [treated 11 (36.67%), control 1 (3.33%)], G3: [treated 1 (3.33%), control 0], G4: [0, 0]	Persistent symptomatic erythema	3
7	Anqi Li (2019) [25].	Clinical trial	308‐nm excimer lamp with minoxidil	34 Patients	21 M,13 F	12 Weeks, twice a week, alopecic patch divided in control and treated side + topical minoxidil into both sides	Initial at a dose of 50 mJ lower than the MED, then adjusted according to the skin reaction	Hair diameter, hair count and G0 no growth, G1 < 25% regrowth (poor), G2 < 50% regrowth (fair), G3 < 75% regrowth (good), G4 complete regrowth (excellent)	G0: [treated 2 (5.8%), control 4 (11.7%)], G1: [treated 3 (8.8%), control 8 (23.5%)], G2: [treated 14 (41.1%), control 14 (41.1%)], G3: [treated 8 (23.5%), control 6 (17.6%)], G4: [treated 7 (20.5%), control 2 (5.8%)] Hair diameter: treated [before 41.38 ± 13.03 in µm, after 74.53 ± 13.87], control [before 42.09 ± 12.30 in µm, after 62.35 ± 15.71], *p* = 0.001 Hair density: treated [before 32.06 ± 18.47/cm^2^, after 123.38 ± 36.34/cm^2^, *p* < 0.001], control [before 31.88 ± 17.54/cm^2^, after 100.88 ± 31.40/cm^2^], *p* = 0.006, Terminal hair count: trated [before 21.94 ± 12.36/cm^2^, after 91.88 ± 39.12/cm^2^], control [before 20.00 ± 11.70/cm^2^, after 75.61 ± 34.26/cm^2^],*p* = 0.079 Vellus hair count: treated [before 10.12 ± 8.31/cm^2^, after 31.50 ± 16.86/cm2], control [before 11.88 ± 8.02/cm^2^, after 25.26 ± 14.90/cm^2^], *p* = 0.094	Hyperpigmentation and erythema, itching, desquamation, and pain	5
8	Yukiyasu ARAKAWA (2016) [26].	Cohort	308‐nm excimer light	11 Patients	1 M, 10 F	At least 8 months, every 2 weeks, irradiating along the midline of the scalp, then temporal regions of the head after sufficient terminal hair regrowth was seen along the midline	Initial dose of 200 mJ/cm^2^, with increments of 50 mJ/cm^2^ in each step	SALT score	From 96.8 ± 10.6 to 31.8 ± 40 (%) no patient with complete remission, the first occurrence of terminal hair growth after a mean of 15.5 ( ± 4.89) sessions	Not reported	6
9	Tzu‐Chien Hsu (2014) [27].	Cohort	A 308‐nm excimer lamp	17 Patients	6 M, 11 F	At least 8 weeks, two to three times each week,	VTRAC™ Excimer Lamp, initial dose of 50 or 100 mJ/cm^2^, with increments of 50 mJ/cm^2^ every week	Response rate is defined as the percentage of patients with regrowth of hair to any extent, a “satisfactory response rate” is defined as the percentage of patients with hair regrowth of more than 50% of the hair loss areas	The overall response rate was 41.1%, and the satisfactory response rate was 29.4%.	Painful erythema, desquamation, pruritus	5
10	Medhat El‐Mofty (2019) [28].	Clinical trial	PUVA	40 Patients	32 M, 8 F	3 Months, twice a week, group A PUVA, group B intralesional corticosteroid (ILC)	Initial dose of 6 or 3 J/cm^2^, with increments of 2 J/cm^2^ every session	SALT score, patient satisfaction	Patient satisfaction 5point scale: 2.7 ± 1.45 (95% CI: 2.02–3.38) SALT score: before (41.47 ± 23.62), after (24.4 ± 20.95 (14.6:34.2))	Pain, hyperpigmentation, blistering, itching, erythema	4
11	Mostafa Mokhtar Kamel (2011) [29].	Clinical trial	PUVA	35 Patients	27 M, 8 F	Every 3 months, max. 1 year	Initial dose of 6 J/cm^2^, with increments of 1 J/cm2 every session	SALT score	Before median 2, IR(1–3), after median 1, IR(0–2), *p* < 0.001	Erythema, burning sensation, and hyperpigmentation	3
12	Kui Young Park (2013) [30].	Cohort	PUVA	32 Patients	19 M, 13 F	16 Weeks, twice a week, concomitant oral cyclosporine	Initial dose of 1 J/cm^2^, with increments of 0.5 J/cm^2^	Excellent response, more than a 50% extent of hair regrowth; good response, hair regrowth of 25%–50%; fair response, hair regrowth of 10%–25%; and poor response, hair regrowth of 0%–10%	3 (9.4%) of excellent responses; 5 (15.6%) of good responses; 11 (34.4%) of fair responses, 13 (40.6%) poor response	Gastrointestinal disturbance, hypertrichosis, headache, and hypertension	5
13	GürolAçıkgöz (2013) [31].	Clinical trial	Topical 8‐MOP plus targeted UVA	7 Patients	7 M	15–24 Sessions, 3 times a week	Daavlin T500x targeted phototherapy device, the initial dose of 0.3 or 0.5 J/cm^2^, with increments of 20%–30% every third therapy session	Four‐point scale (0 = no hair, 1 = white vellus hair, 2 = regrowth cosmetically acceptable for the patient, 3 = complete hair growth)	Score 1: 3, score 2: 1, score 3: 3, *n* = 7	Slight erythema	3
14	Lucinda Siyun Tan (2020) [32].	Clinical trial	PUVA	10 patients	6 M, 4 F	21–151 sessions, twice a week	With a curve panel and a flat panel UVA unit, the initial dose of 2.88–4.88 J/cm^2^	Percentage of terminal hair regrowth and percentage change in baseline severity of alopecia tool (SALT) score	All the patients who continued treatment with paint PUVA without premature termination were successful in hair regrowth to more than 75%.	Scalp tenderness	3
15	Maira E. Herz‐Ruelas (2017) [33].	Clinical trial	UVA‐1	22 Patients	9 M, 13 F	6 Months, 3 to 5 times a week	Sellamed 3000 UVA‐1‐Part‐body Radiation System, 25 sessions of 30 J/cm^2^, then 60–120 J/cm^2^	SALT score defined as S0‐absence of hair loss, S1‐less than 25%, S2‐hair loss between 25% and 49%, S3‐hair loss between 50% and 74%, S4‐hair loss between 75% and 99%, and S5‐100% hair loss	Mean difference in SALT absolute value: in 30 J/cm^2^ (−1.09 ± 4.02, *p* = 0.21), in 60 J/cm^2^ (−2.6 ± 5.05, *p* = 0.02), in 120 J/cm^2^ (−1.42 ± 3.73, *p* = 0.08)	Mild xerosis, mild hyperpigmentation	3
16	Muhsin A. Al‐Dhalimi (2019) [34].	Clinical trial	1540 nm Fractional erbium‐ glass laser	30 Patients (60 patches)	18 M, 12 F	6 Weeks, every week, patches were divided to study and control groups, both receiving topical minoxidil	Quanta System S.P.A. DNA Laser Technology, 1 Hz frequency, 7 ms pulse duration, spot size 8 mm, energy MTS (microthermal spot) as 8.1 mJ, with 300 μm estimated depth	Hair count, percentage of hair regrowth, patient satisfaction	Hair count: before: (8.4667 ± 4.63669), after: (9.3000 ± 4.67680), follow‐up: (10.9667 ± 4.83153), *p* = 0.001, Percentage of hair growth: before: (21.83 ± 13.8), after: (26.91 ± 15.61), follow‐up: (31.11 ± 18.40), (*p* = 0.002), patient satisfaction: after: (2.600 ± 0.968) vs. (2.533 ± 1.136) in the control group,(*p* = 0.808), follow‐up: (4.766 ± 1.755) vs. (2.866 ± 1.195),(*p* = 0.001)	Burning sensation, erythema, itching	4
17	Wuyuntana Wang (2019) [35].	Clinical trial	1550‐nm NAF erbium–glass laser	8 Patients	4 M, 4 F	20 Weeks, every 2 weeks	GSD, 10–15 mJ of energy with a total intensity of 300 spots/cm^2^	Score: 0, no effect; 1, hair regrowth involving < 50% of the lesions; 2, hair regrowth involving more than 50% of the lesions	Score 0: 2, score1: 1, score2: 5, *n* = 8	Mild erythema and mildly broken hair shafts	3
18	Ji Hae Lee (2020) [36].	Clinical trial	311‐nm Titanium: Sapphire laser (TSL)	19 Patients	9 M, 10 F	Once or twice weekly	Gain‐switched 311‐nm TSL, the initial dose of 300 mJ/cm^2^, with increments of 50 mJ each session until MED	No regrowth, 0%; mild, 1%–24%; moderate, 25%–49%; good, 50%–74%; excellent, 75%–99%; complete, 100%	14 Patients (73.9%) excellent to complete, 1 patient marked and 2 moderate, 2 patients no response	Persistent erythema	3
19	BasakYalici‐Armagan (2016) [37].	Clinical trial	Long‐pulsed neodymium: yttrium aluminum garnet (Nd: YAG) laser and fractional carbon dioxide laser	32 Patients (96 patches)	19 M, 13 F	Nd: YAG laser 2–3 sessions with 2–8‐week intervals, the fractional carbon dioxide laser 3–6 sessions with 2 or 4‐week intervals, patchANd: YAG laser, patch B CO_2_ laser, patch C control	LaserscopeLyrai, shortest pulse duration of 30 ms and lowest energy of 10 J/cm^2^, eCO_2_, power of 30 W, 120 mm probe diameter, pulse energy 10–45 mJ/cm^2^, and density 75–100 spot/cm^2^/pass	SALT score, hair count	SALT score: Nd‐YAG: initial median = 28, final median = 33, *p*‐value = 0.4 fractional CO_2_: initial median = 28, final median = 33, *p*‐value = 0.4 Hair count: Nd‐YAG: before (58.38 ± 27.42), after (62.41 ± 27.88), *p*‐value = 0.4 fractional CO_2_: before (64.25 ± 23.08), after (70.69 ± 27.16), *p*‐value = 0.17	Pain in the CO_2_ laser group	5
20	Rania El‐Husseiny (2020) [38].	Clinical trial	Fractional CO_2_ laser	20 Patients	12 M, 8 F	3 Months, every 2 weeks, one patch treated with FCO_2_ laser, the other with ILC (Triamcinolone)	Fire‐Xel Bison laser, pulse width: 1026‐1346 μ, density: 0.8, and energy: 30.7–40.3 mJ	Patient satisfaction (10 point VAS), hair count, < 25% = no or minimal improvement, 25%–49% = moderate, 50%–74% = marked, > 75%– 99 = excellent, and 100% = complete improvement	CO_2_: 2/20 minimal, 3/20 moderate, 3/20 marked, 7/20 excellent, Median 4 ( > 75%–99%), IQR 2.5–4.5, ILC: 11/20 minimal, 9/20 moderate, Median 1 ( < 25%), IQR 1–2, *p*‐Value < 0.001, hair count: CO_2_: Median = 39.5, IQR = 16–68 (Hair/cm2), ILC: Median = 11.5, IQR = 6–22, *p*‐Value < 0.001, patient satisfaction: CO_2_: Median = 9, IQR = 7.5–10, ILC: Median = 3, IQR = 1–4, *p*‐Value < 0.001	Mild pain, transient posttreatment scaling, erythema, and edema	4
21	Imran Majid (2018) [39].	—	Fractional CO_2_ laser	8 Patients	4 M, 4 F	Max. 8 sessions, every 3 weeks, spraying triamcinolone solution immediately after each session	120 µm tip, fluence of 50–60 mJ/cm^2^, and density of 100 microthermal zones (MTZ)/cm^2^	Percentage of hair regrowth	7 Patients with more than 75% regrowth	Nothing reported	—
22	Nermeen Mohamed Abdelhalim (2014) [40].	Clinical trial	LLLT	23 Patients (52 patches)	14 M, 9 F	4 Weeks, 3 times a week, 23 patches as control, 29 patches treated	ENDOLASER 422 – Enraf‐Norius® with a dose of 1.5 J/cm², 905 nm wavelength, and 5000 Hz frequency with a peak power of 100 W	hair count and visual analog scale (VAS) of hair loss (0–100)	VAS: before (87.79 ± 7.64), after (40.94 ± 26.92), Follow‐up (26.1 ± 31.33), *p*‐value = 0.001, hair count: before (18.79 ± 8.84), after (37.93 ± 16.48), Follow‐up (41.48 ± 17.3), *p*‐value = 0.001	Not reported	3
23	HEBA A. EID (2018) [41].	Clinical trial	Polarized (Biopteron) light therapy	30 Patients	—	3 Months, 3 times a week, Group A treated, Group B control, both receiving topical minoxidil	B‐type lens unit at a setting of 1.26 W, with 4‐s pulses delivered at 1‐s intervals	Hair count, the 7‐point scale for global photos assessment	7‐Point scale: treated (before: −3, after:+1, *p* = 0.0001), control(before: −3, after: −1, *p* = 0.0001), *p* = 0.0001, hair count: treated(before: 0 ± 0, after:107.86 ± 20.6, *p* = 0.0001), control(before: 0 ± 0, after: 64.46 ± 5.08, *p* = 0.0001), *p* = 0.0001,	Not reported	4
24	KWANG HO YOO (2010) [42].	Clinical trial	Photodynamic therapy (PDT) with red light	8 Patients	2 M, 6 F	3 Sessions, every 4 weeks, microneedle rolling on the right side of the scalp, red light on both sides of the scalp	Aktilite lamp, average wavelength, 630 nm; light dose, 37 J/cm^2^	Hair regrowth	No hair regrowth on both sides	Mild pain and erythema	3
25	C. M. Giorgio (2019) [43].	Clinical trial	Photodynamic therapy (PDT)	41 Patients	17 M, 24 F	18 Weeks, once every 3 weeks, Group A microneedling, Group B ALA‐PDT, group C both micro‐needling and ALA‐PDT	630 nm, 37 J/cm^2^	0, no hair regrowth; 1, < 50% hair regrowth; 2, ≥ 50% hair regrowth; and 3, complete (100%) hair regrowth	Group A: no hair regrowth, Group B: 0(4), 1(1), 2(5), 3(2), Group C: 0(1), 1(6), 2(7), 3(3)	Not reported	3
26	Ali Tanakol (2020) [44].	Clinical trial	2940‐nm erbium:yttrium‐aluminum‐garnet (Er:YAG) laser	25 Patients (16 scalp AA)	19 M, 6 F	3 Sessions, 4–6 weeks apart,	Fotona, the spot size of 4 mm, pulse energy 1.2 J, one laser pass, frequency of 6 Hz, long‐pulsed mode	Hair regrowth, SALT score, patient satisfaction (0–5)	Mean regrowth rate of 17.4 ± 3, 5% (0–100), A total of 16 patients who were treated for patchy alopecia areata of the scalp showed 27.8 ± 31.3% regrowth. mean percent change in the SALT score: 17.4 ± 3.5%, patient satisfaction: 1.84 ± 2.21	Transient erythema, folliculitis	4

Abbreviations: AA, alopecia areata; LLLT, low‐level light/laser therapy; PUVA, psoralen ultraviolet A; UVA, ultraviolet A; UVB, ultraviolet B.

**Table 3:** Summary of results in androgenic alopecia.

	First author, year	Study type	Light/laser type	Sample size	Sex	Intervention	Laser/light features	Judgment criteria	Results	Side effect	Risk of bias
1	Mohammed K. Alhattab (2019) [45].	Clinical trial	1540‐nm fractional erbium‐glass laser	47 Patients	22 M, 25 F	5 months, every 2 weeks	Quanta System SPA DNA Laser Technology, 7 mm tip, 6 mJ pulse energy, 1 HZ frequency	Hair diameter, hair count, patient satisfaction, global evaluation scale (−3, +3)	Global scale: 12/47 + 1, 13/47 + 2, and 2/47 + 3, 18/47 0, and 2/47 ‐1, hair diameter: Women: (before 3.12 ± 0.43), (after 3.84 ± 0.51), *p*‐value = 0.001, Men: (before 2.68 ± 0.56), (after 3.29 ± 0.7), *p*‐value = 0.001, Hair count: Women: (before 72.8 ± 21.58), (after 83.08 ± 27.32), *p*‐value = 0.001, Men: (before 59.36 ± 16.46), (after 66.68 ± 19.91), *p*‐value = 0.001, patient satisfaction: 17/47 mild satisfaction, 14/47 moderate satisfaction, and 4/47 excellent satisfaction; 12/47 not satisfied (stabilized), and no one became worse,	Mild erosion, mild erythema, burning sensation	4
2	JitladaMeephansan (2018) [46].	Clinical trial	1550 nm Er: Glass fractional laser	23 Patients	16 M, 7 F	24 Weeks, every 2 weeks,	MOSAIC, 2*12‐mm tip, power 6 mJ, spot density 300 spots/cm^2^ (static mode), two passes	Hair diameter, terminal hair count, non‐vellus hair count	Hair diameter: before (42.52 ± 9.82 μm), after (50.74 ± 10.69), *p* = 0.027, Terminal hair count: after (93.91 ± 29.96 per cm^2^) vs. before (70.43 ± 26.88 per cm^2^); *p* = 0.001, non‐vellus hair count: before (159.13 ± 28.91), after (177.39 ± 37.2 per cm^2^); *p* = 0.061,	TTolerable pain, significant itching, mild erythema	3
3	G.‐Y. Lee (2011) [47].	Clinical trial	1550 nm Er: Glass fractional laser	27 Patients	27 F	5 Months, every 2 weeks	Mosaic, 5–10 mm tip, 6;mJ pulse energy, 800 spot ⁄ cm^2^ density, static mode	Hair diameter, hair count	Hair diameter: before 58 ± 12, after 75 ± 13 μm (*p* < 0.001), Hair count: before 100 ± 14 ⁄ cm^2^, after 157 ± 28 ⁄ cm^2^ (*p* < 0.001),	Pruritus	3
4	PoonkiatSuchonwanit (2019) [48].	Clinical trial	1550 nm Er:Glassfractional laser	29 Patients	29 M	24 weeks, every 2 weeks, one side of scalp monotherapy with topical minoxidil, the other side combined laser and minoxidil therapy	Fines can, 7 mm probe diameter; energy 6 mJ; density 300 spot/cm^2^; 10% overlapping treatment area	Hair diameter, hair count, 7‐point global assessment scale, as follows (−3, +3)	Hair diameter: treated: [from 50.93 ± 13.59 to 67.28 ± 15.63 μm, *p* = 0.001], control: [from 51.16 ± 14.53 to 65.32 ± 16.42 μm, *p* = 0.002], *p* = 0.03, Hair count: treated: [before 96.58 ± 16.52, after 147.12 ± 18.19 hairs/cm^2^, *p* = 0.001], control: [before 97.25 ± 15.91, after 133.77 ± 19.42 hairs/cm^2^, *p* = 0.001], *p* = 0.004, 7‐point scale: treated [0(0), +1(1), +2(12), +3(16)], control [0(0), +1(3), +2(19), +3(7)],*p* = 0.04,	Tolerable pain, warmth sensation, erythema, itchiness, scaling	4
5	WON‐SERK KIM (2010) [49].	Clinical trial	1550 nm Er: Glass fractional laser	20 Patients	20 M	10 Weeks, every 2 weeks, the right side of the scalp is the treated side, and the left side is the control	Energy 5 mJ, the density of 300 spots/cm^2^	Hair density, hair caliber	Significant increase in hair density but no significant increase in hair caliber, 4 months after the final treatment density decreased to baseline level.	Pain, transient shedding, erythema, pruritus, dryness, dandruff	3
6	Doris Day (2021) [50].		Nonablative Er:YAG laser	16 Patients	7 M, 9 F	16 Weeks, every 2 weeks, In 10 out of 16 patients, treatment was combined with PRP at every other session	SP Dynamis, Fotona, 2940 nm, 7 mm spot size, 7.00 J/cm^2^ pulse fluence, 3.3 Hz frequency	Clinical and blind evaluation	Reduction of AGA grade compared to baseline (*p* = 0.015 and *p* = 0.125 in female and male patients, respectively)	Pain	3
7	Yue Huang (2017) [51].	Clinical trial	Ablative carbon dioxide (CO_2_) fractional laser	27 Patients	27 M	6 Sessions, every 2 weeks, one side of the scalp treated, the other side left as control, topical hair growth factor on the entire scalp	Pixel CO_2_, 50‐mm tip, 12–18 mJ/spot, 361 spots/cm^2^, one pulse, and 40% density	7‐Point global‐assessment scale (−3, +3), hair diameter, hair count	(+1, +2, +3) 25/27 (93%), (0) 2/27 (7%), (−1, −2, −3) 0, *p* = 0.009, Hair diameter: treated: before (44.32 ± 3.89 μm), after (58.39 ± 9.29 μm), control: 44.32 ± 6.04 to 54.74 ± 7.88 μm, Hair count: treated: before (114 ± 27/cm^2^), after (143 ± 25/cm^2^), *p* = < 0.001 control: before (113 ± 24/cm^2^), after (134 ± 19/cm^2^), *p* < 0.001, comparison *p* = 0.002,	Erythema, edema, pruritus, dryness, seborrheic dermatitis, and dandruff	4
8	Sung Bin Cho (2018) [52].	Clinical trial	1927‐nm fractionated thulium laser	10 Patients	10 M	12 Weeks, every week, GF solution on a randomly half a	LASEMD™, power of 5 W and energy of 4 mJ (50.9 J/cm^2^) or 6 mJ (76.4 J/cm^2^), static operating mode	Hair diameter, Hair count	Hair diameter: before (0.047 ± 0.009 mm), after (0.059 ± 0.01 mm), (*p* < 0.001), hair count: before (163.7 ± 31.1), after (195.3 ± 34.3), (*p* < 0.001)	Mild transient scalp redness, mild itching, mild burning sensation, mild to moderate seborrheic dermatitis	4
9	Sung Bin Cho (2016) [53].	Clinical trial	1927‐nm fractionated thulium laser	16 Patients	12 M, 4 F	12 weeks, every week, group A: Thulium laser and PDRN, group B: mesotherapy and PDRN	LASEMD™, power of 5 W, energy of 6 mJ under a static operating mode	Hair thickness, hair count	Thickness: before (4.038 ± 1.678), after (5.906 ± 1.946), *p*‐value: < 0.001, count: before (67.875 ± 15.16), after (80.875 ± 16.924), *p* = 0.005	Itching sensations and desquamation	3
10	Joaquin J. Jimenez (2014) [54].	Clinical trial	A low‐level laser device	225 Patients	103 M, 122 F	26 Weeks, 3 times a week	HairMax Laser Comb, 7‐ and 9‐beam lasercombs655 nm, 12‐beam wavelength of 635 nm	Hair count	Female (9‐beam laser comb: (before 162.6 ± 46.2, change after 16w 14.8, after 26w 20.2, *p* < 0.0001), 12‐beam laser comb: (before 142.2 ± 40.5, after 16w 11.9, after 26w 20.6, *p* < 0.0001), male (7‐beam laser comb: (before 211.5 ± 54.0, change after 16w 17.7, after 26w 18.4, *p* = 0.0017), 9‐beam laser comb: (before 163.3 ± 69.4, change after 16w 20.4, after26w 20.9, *p* = 0.0249), 12‐beam laser comb: (before 151.5 ± 42.4, change after 16w 23.5, after 26w 25.7, *p* = 0.0028)	Dry skin, pruritus, scalp tenderness, irritation, warm sensation	5
11	Jung Soo Yoon (2020) [55].	Clinical trial	Low‐level light therapy (LLLT)	59 Patients	39 M, 20 F	16 Weeks, every other day, group A: experimental device, group B: sham device	HAIRUP, 2.36 mW/cm^2^ (2.56 mW/cm2) at a wavelength of 655 nm	Hair count, hair diameter, patient satisfaction	Diameter: before: [treated 62.20 ± 23.23, sham 68.86 ± 21.22, *p* = 0.255], after: [treated 7.50 ± 10.36, sham ‐15.03 ± 9.94, *p* < 0.001], Count: before: [treated 109.27 ± 32.75, sham 113.62 ± 29.86, *p* = 0.596], after: [treated 41.90 ± 23.77, sham 0.72 ± 15.39, *p* < 0.001], patient satisfaction(0‐10): Week8: [treated 36.30 ± 29.04, sham 26.38 ± 22.63, *p* = 0.169], Week16: [treated 43.17 ± 31.52, sham 35.14 ± 26.11, *p* = 0.265]	Nothing reported	5
12	Poonkiat Suchonwanit (2018) [56].	Clinical trial	LLLT	36 Patients	19 M, 17 F	24 Weeks, 3 times a week, 19 patients in laser and 17 patients in the sham group	RAMACAP, 660 ± 10 nm, the power density of 3.5 mW/ cm^2^, fluence of 4 J/cm^2^	Hair density, hair diameter	Diameter: treated [Baseline (53.8 ± 11.6), mean change 6.11 ± 2.15 μm], sham [Baseline (54.2 ± 15.1), mean change 3.76 ± 1.24 μm], *p* = 0.009, Density: treated [Baseline (112.4 ± 18.9), mean change 10.21 ± 3.25 hairs/cm^2^], sham [Baseline (115.4 ± 16.7), mean change 3.95 ± 1.32 hairs/cm^2^], *p* = 0.002,	Increased hair shedding, mild scalp itching	4
13	Hyojin Kim (2013) [57].	Clinical trial	LLLT	40 Patients	26 M, 14 F	24 Weeks, 18 min daily, sham group, and treated group	The Oaze, 60.7 mW/cm^2^ for the 630‐nm LED, 182.8 mW/cm^2^ for the 660‐nm LED, and 115.4 mW/cm^2^ for the LD; total energy density of 92.15 mW/cm, Energy fluence 47.90 J/cm^2^	Hair diameter, hair count, global assessment of hair regrowth (0–4)	G0= (sham2/14, LLLT0/15), G1= (sham11/14, LLLT7/15), G2 = (sham1/14, LLLT4/15), G3 = (sham0/14, LLLT3/15), G4 = (sham0/14, LLLT1/15), n = 29, *p* = 0.002, Diameter: before (56.2 ± 17.9), after (68.8 ± 13.2), sham (3.9 ± 7.3), LLLT (12.6 ± 9.4), *p*‐value = 0.01, Count: before (117.2 ± 19.6), after (134.4 ± 20.2), *p*‐value = , sham (–2.1 ± 18.3), LLLT (17.2 ± 12.1), *p*‐value = 0.003	Headache, skin pain, pruritus, erythema, and acne	4
14	Sabrina Mai‐Yi Fan (2018) [58].	Clinical trial	LLLT	74 Patients	61 M, 13 F	24 Weeks, 3 times a week, half of the scalp received LLLT, the other half sham light	The iRestore ID‐520, 660 nm with 22 mW/cm^2^, or 659 nm with 4.6 mW	Investigator's global assessment (IGA), hair coverage, hair diameter, hair count	IGA: Week4 (1.2 ± 0.6), week12 (1.7 ± 0.6),*p* < 0.001, Week24(2.0 ± 0.6), *p* < 0.001, Hair coverage: before (10.9% ± 5.9), after(12.9 ± 6.5), *p* < 0.001, Diameter: before (33.1 ± 8.1), change at Week 24 from baseline (1.9 ± 4.4), *p* < 0.001, Count: before (98.5 ± 23.8), change at w24 from baseline (6.0 ± 12.5), *p* < 0.001,	Eczema, pruritus, and acne	4
15	BehroozBarikbin (2017) [59].	Clinical trial	Low‐level diode laser scanner	90 Patients	60 M, 30 F	Max. 4 months, 3 times a week, group 1: 655 nm with 3 J/cm2, group 2: 655 nm with 2 J/cm2 plus 808 nm with 1 J/cm2, group 3: control group	Laser scanner, 655 nm, and 808 nm	Hair count, patient satisfaction (G1–G4)	Patient satisfaction: Laser scanner: (end G4:2 G3:19 G2:11, 6 m F/U: G4:1 G3:6 G2:22 G1:1, 12 m F/U: G4:1 G3:5 G2:23 G1:1), Laser hat:(end G4:1 G3:17 G2:10 6 m G1:2, F/U: G3:8 G2:20 G1:2, 12 m F/U: G3:5 G2:21 G1:4), Count: Laser scanner: (before 52.06 ± 48.51, after 61.67 ± 53.11, *p*‐value 0.0001), Laser Hat: (before 75.07 ± 54.58, after 84.23 ± 57.69, *p*‐value 0.0001), sham laser: (before 72.29 ± 57.39, after 70.48 ± 56.24, *p*‐value 0.003),	Mild headache, dry skin	4
16	Gita Faghihi (2018) [60].	Clinical trial	LLLT	45 Patients	15 M, 30 F	6 Months, 2 or 3 sessions per week, 22 patients received sham light, 23 received LLLT, both groups treated with topical minoxidil	With a 10–50 MW power and a 785‐nm wavelength	Hair diameter, hair count, patient satisfaction	Diameter: before (case 0.050 ± 0.006, control 0.051 ± 0.011 *p* = 0.87), after 6 months (case 0.057 ± 0.005, control 0.054 ± 0.012, *p* = 0.43), after 9 months (case 0.064 ± 0.006, control 0.061 ± 0.013, *p* = 0.03), after 12 months (case 0.073 ± 0.006, control 0.067 ± 0.015, *p* = 0.045), Count: before (case 14.9 ± 2.8, control 14.8 ± 2.9, *p* = 0.95), after 6 months (case 17.4 ± 2.7, control 16.5 ± 3.1, *p* = 0.26), after 9 months (case 23.04 ± 3.3, control 19.2 ± 3.3, *p* < 0.001), after 12 months (case 25.9 ± 2.5, control 22.2 ± 3.8), patient satisfaction: after 6 months: High (case 26.1%, control 0, *p* < 0.001), Moderate (case 73.9%, control 4.5%), Low (case 0, control 72.8%), Zero (case 0, control 22.7%), after 12 months: Very high (case 43.5%, control 0), High (case 56.5%, control 4.5%), Moderate (case 0, control 41%), Low (case 0, control 50%),	Headache, itching, burning, erythema	4
17	Yang Liu (2020) [61].	Clinical trial	LLLT	83 Patients	83 F	6 Months, every other day, Group A: LLLT, Group B: Minoxidil, group C: Both	iHelmet, 200 5 mW laser diode source (650 nm) arrays	Hair count, hair diameter, scalp oil secretion	Diameter: before (49.89 ± 6.349), after (62.39 ± 6.932), *p* < 0.001, Count: Before (79.32 ± 5.432), after (104.82 ± 5.386), *p* < 0.001, scalp oil secretion: before (68.71 ± 11.885), after (53.57 ± 11.230), *p* = 0.028,	Scalp tenderness, mild scalp pruritus	3
18	Raymond J. Lanzafame (2014) [62].	Clinical trial	LLLT	42 Patients	42 F	16 Weeks, every other day, 24 patients in treated, and 18 patients in the sham group	TOPHAT655, 655 nm with 7 J/cm^2^ irradiance and 2.9 J dose	Hair count	Case: [before (335.4 ± 144.6), after (157.8 ± 50.5), *p* = 0.019], sham: [before (317.5 ± 174.1), after (183.5 ± 84.9), *p* = 0.125] *p* = 0.846 and 0.361,	Nothing reported	3
19	Shelly Friedman (2017) [63].	Clinical trial	LLLT	40 Patients	40 F	17 Weeks, every other day, 19 patients in treated, and 21 patients in the sham group	Capillus272 Pro device, 650 nm, 1360 mW total delivered energy over 582 cm^2^ or 2.34 mW/cm^2^	Hair count	Terminal hair count: before (189.3 ± 85.8), after (268.3 ± 117.7), *p* < 0.001. Subjects receiving LLLT achieved a 51% increase in hair counts as compared with sham‐treated control patients. (LLLT: 63.67 ± 50.9, sham: 12.48 ± 13.76, *p* < 0.001).	Nothing reported	3
20	Samia M. Esmat (2017) [64].	Clinical trial	LLLT	45 Patients	45 F	4 Months, 3 days weekly, group 1: topical minoxidil, group 2: LLLT, group 3: both	iGROW1 helmet device, < 5 mW CW and LED wavelength range from 650 to 670 nm	Patient satisfaction (0 = dissatisfied, 3 = satisfied), hair diameter, hair count	Patient satisfaction: 3/15 score 0, 7/15 score 1, 4/15 score 2, 1/15 score 3, Diameter: before (0.047 ± 0.013), after 2m (0.051 ± 0.017), *p*‐value = 0.099, after 4m (0.063 ± 0.023), *p* = 0.118, Count: before (145.47 ± 20.37), after 2m (146.07 ± 19.76), *p*‐value = 0.346, after 4m (195.53 ± 14.71), *p* < 0.001,	Irritation, scalp tenderness, warm sensation, the initial increase in the hair shedding	4
21	Raymond J. Lanzafame (2013) [65].	Clinical trial	LLLT	41 Patients	41 M	16 Weeks, every other day, 22 patients in active, and 19 patients in the sham group	TOPHAT655, 5 mW lasers (655 ± 5 nm), and 31 LEDS (655 ± 20 nm), 67.3 J/cm^2^ irradiance	Hair count	Case: before (142.0 ± 73.0), after (228.7 ± 102.8), sham: before (162.7 ± 95.9), after (162.4 ± 62.5), *p* = 0.426 and 0.016,	Nothing reported	3
22	Kenneth Blum (2014) [66].		Cold beam low‐level laser light	119 (48 subjects completed all study visits)	119 M	26 Weeks, 70 patients as case and 49 as control	The novel cold X5 hair laser device,	Hair count	Baseline (159.00 ± 65.39), Week4 (167.90 ± 63.55), Week8 (169.30 ± 71.24), Week14 (178.90 ± 69.63), Week20 (170.00 ± 67.46), Week26 (174.80 ± 68.97), *p*‐value < 0.0001,	Nothing reported	4
23	AndréiaMunck (2014) [67].	Clinical trial	LLLT	32 Patients	11 M, 21 F	8.7 ± 5.2 months, 3 times weekly	HairMax Laser Comb, 655 nm	Global photographic assessment	8 Patients showed significant, 20 moderate, and 4 no improvement	Nothing reported	3
24	Mohamed Amer(2021) [68].	Clinical trial	LLLT	13 Patients	13 F	16 Weeks, 2 sessions per week,	iGrow Hair Growth System (TOPHAT655), 655 nm	Hair count	Before 222.3 ± 33.5, after 255.3 ± 30.4, *p* = 0.007	Headache, dryness of hair, heat sensation, scalp tenderness	4
25	Fernanda Ferrara (2021) [69].	Clinical trial	Low‐level laser irradiation	19 Patients	19 M	6 Months, 2 times per day, the left half of the device emitted light, and the right half did not, topical minoxidil on both sides	Capellux®, 5 mW of 660 nm light, a total of 5.5 J/cm^2^/day	Hair count	Total hair count: sham (80.2 ± 20.3 to 110.3 ± 22.6, *p* < 0.001), active (76.3 ± 20.1 to 100.8 ± 25.1, *p* = 0.001), terminal hair count: sham (55.7 ± 22.8 to 62.1 ± 25.5, *p* = 0.089), active (49.3 ± 21.5 to 57.6 ± 21.2, *p* = 0.012), vellus hair count: sham (26.2 ± 21.6 to 48.1 ± 26.8, *p* < 0.001), active (27.0 ± 17.7 to 43.2 ± 26.1, *p* = 0.016),	Nothing reported	4
26	G. Lodi (2021) [70].		Blue LED light	20 Patients	20 M	10 Weeks, twice a week,	Blue LED light device, 417 ± 10 nm, fluence of 120 J/cm^2^, and power intensity of 60 mW/cm^2^ ± 20%	Hair density and hair shaft width	Density: (106 ± 66 to 117 ± 69 units/cm^2^, *p* = 0.001), Shaft width: (0.0295 ± 0.017 to 0.034 ± 0.017 mm, *p* = 0.009)	Slight darkening of hair	3

**Table 4:** Summary of results in cicatricial alopecia.

	First author, year	Study type	Alopecia type	Light/laser type	Sample size	Sex	Intervention	Laser/light features	Judgment criteria	Results	Side effect	Risk of bias
1	Pablo Fonda‐Pascual (2018) [71].	Clinical trial	Lichen planopilaris (LPP)	Low‐level light therapy (LLLT)	8 Patients	3 M, 5 F	6 Months, daily	Skymedic, 630 nm and a fluence of 4 J/cm^2^	Lichen planopilarisactivity index (LPPAI), terminal hair thickness (THT)	LPPAI (0–10): before mean 3.35 (range 2.50‐4.50), after 3 m mean 2.417 (range 1–7.5), *p* = 0.161, after 6 m mean 0.865 (range 0–2.0), *p* = 0.012, THT: before mean 71.7 (range 60.0–89.0), after 3 m mean 105.57 (range 100.0–111.0), *p* = 0.018, after 6 m mean 82.4 (range 78.0–89.0), *p* = 0.035	Not reported	4
2	AgnieszkaGerkowicz (2019) [72].	Clinical trial	frontal fibrosing alopecia (FFA) and lichen planopilaris (LPP)	novel superluminescent light‐emitting diodes (sLEDs)	16 Patients (8 with FFA and 8 with LPP)	16 F	10 Weeks, weekly	Red Beam Pro + 630 ± 5 nm with a maximum power density of 100–120 mW/cm^2^, dose per session 44.45 J/cm^2^ and light power density of 70 mW/cm^2^	Lichen Planopilaris Activity Index (LPPAI) 1‐10, Frontal Fibrosing Alopecia Severity Score (FFASS) 0‐25	LPPAI: before (median 4.66, IR 3.23), after (median 1.33, IR 1.88), *p* = 0.012, FFAS: before (median 10.85, IR 6.25), after (median 10.25, IR 6.00), *p* = 0.017	Stinging sensation, pleasant warmth, and skin dryness	4
3	SUHYUN CHO (2013) [73].	Clinical trial	Ophiasis (*n* = 3), autosomal recessive woolly hair/hypotrichosis (*n* = 3), secondary cicatricialalopecias (pressure‐induced alopecia, *n* = 3; scleroderma, *n* = 2; surgical scar, *n* = 2), pubic hypotrichosis (*n* = 2), frontal fibrosing alopecia (*n* = 1), and perifolliculitisabscedens et suffodiens (*n* = 1).	1550‐nm erbium – glass laser (NAFL), CO_2_ laser (AFL)	17 Patients	9 M, 8 F	8–22 sessions (mean = 15.2), every 2–4 weeks	For NAFL fluence of 6–8 mJ and a density of 300 spots/cm^2^/pass, for AFL fluence of 30–50 mJ, a density of 150 spots/cm^2^	Global improvement score scale (0–4), patient satisfaction (1–4)	Mean clinical improvement score = 2.2, patient satisfaction score = 2.5, 12 (70.6%) demonstrated a clinical response	Pain, transient posttreatment crusting, scaling, erythema, and edema	3
4	Michael J. Randolph (2020) [74].	Case series	LPP	LLLT	4 Patients	4 F	6–18 months, daily	Tricoglam or CapillusPro or Capellux I9, 650 nm wavelength 5 mW power per light or 650nm wavelength with 1360 mW or 660 nm wavelength 25.5 mW/cm^2^ irradiance	Clinical assessment	All patients had improvement of symptoms and signs of disease on dermoscopy after 3 months.	Nothing reported	—
5	Lun Yang (2021) [75].	Clinical trial	Folliculitis decalvans	5‐aminolevulinic acid photodynamic therapy (ALA‐PDT)	13 Patients	13 M	3 Sessions, every 10–14 days	633 nm, intensity of 100 mW/cm^2^	Recovery (symptoms disappearance), significant improvement (lesion reduction> =70%), moderate improvement (lesion reduction > =30%), ineffective (lesion reduction < 30%)	4 Recovered, 7significantly improved, 2 moderately improved	Not reported	3

**Table 5:** Summary of results in telogen effluvium.

First author, year	title	Light/laser type	Sample size	sex	intervention	Laser/light features	Judgment criteria	results	Side effect	Risk of bias
Mohamed Amer (2021) [68].	Clinical trial	Low‐level light therapy (LLLT)	7 Patients	7 F	16 Weeks, 2 sessions per week	TOPHAT655, 655 nm	Hair count	271.2 ± 39.0 vs. 294.2 ± 38.1, *p* = *p* = 0.143	Headache, dryness of hair, heat sensation, scalp tenderness	4

In the alopecia areata domain, 26 articles met the inclusion criteria. Four articles focused on narrow‐band ultraviolet B (NBUVB), five on the 308‐nm excimer laser/light, and five on PUVA therapy. In the androgenic alopecia domain, out of 26 included articles, 17 articles discussed LLLT, and five focused on erbium‐glass laser treatments. Five articles addressed cicatricial alopecia and one dealt with telogen effluvium.

### 3.2 Study Characteristics

The effect of NBUVB was assessed among 161 patients with alopecia areata (AA), including 88 males and 73 females. The initial NBUVB dose ranged from 200 to 300 mJ/cm², administered twice or thrice a week. One study compared NBUVB with topical calcipotriol, while another compared it to intralesional corticosteroid injections. The 308‐nm excimer laser effect was evaluated in 100 patients (54 males and 46 females), with treatment administered twice a week for at least 8 weeks; initial doses ranged from 200 to 400 mJ, increasing based on skin reactions. Four of the five studies employed a self‐controlled design by treating only one side of the alopecic patch. One study combined treatment with topical minoxidil.

Five studies evaluated PUVA therapy among 124 patients (91 males and 33 females), with treatment intervals varying from twice a week to every 3 months. In one study, patients received both oral cyclosporine and lower doses of UVA irradiation; another compared intralesional corticosteroid injections with PUVA therapy. Further details can be found in Table 2.

For androgenetic alopecia (AGA), the erbium‐glass laser was studied in 146 patients (87 males and 59 females). Four of the five studies utilized the 1550‐nm erbium glass laser, while one employed the 1540‐nm variant. Treatment sessions were conducted biweekly, lasting between 5 and 6 months. Overall laser energy was 6 mJ, with a total density of 300 spots/cm². One study combined erbium glass laser with topical minoxidil.

Twenty‐six patients (22 males and 4 females) received treatment with a 1927‐nm fractionated thulium laser, administered weekly for 12 weeks. In one study, half of the scalp was treated with a topical growth factor solution, while another compared thulium laser treatment with mesotherapy. The LLLT effect on AGA was evaluated in 919 patients (490 females, 429 males). Five of the 17 studies specifically targeted female pattern hair loss, while four focused on male pattern hair loss. Treatment protocols varied, with frequencies including three times a week, every other day, and twice a week. Session duration ranged from 8 to 30 min. Study characteristics can be found in Table 3.

For cicatricial alopecia, LLLT was evaluated among 28 patients (25 females and 3 males), with 20 having lichen planopilaris and eight frontal fibrosing alopecia. Variable intervention sessions ranged from daily treatments of 15 min for 6 months to weekly sessions lasting 13 min for 10 weeks. The wavelengths administered ranged from 625 to 660 nm. In patients with telogen effluvium, LLLT was administered twice a week for 16 weeks using TOPHAT655, which emits 655 nm light. Additional details are provided in Table 4.

### 3.3 Study Outcomes

Out of four studies assessing NBUVB on AA, two reported NBUVB as an unsuitable alternative for monotherapy compared to intralesional corticosteroids. However, another study with a larger sample size and extended treatment duration demonstrated a significant decrease in the Severity of Alopecia Tool (SALT) score from 3.97 ± 0.7 to 2.33 ± 0.7 (*p* < 0.001).

The 308‐nm excimer laser was identified as a viable alternative for both monotherapy and adjunctive therapy, significantly improving hair density and diameter, particularly in patients with shorter disease duration and smaller lesions. The mean efficacy of this treatment was 40%, with a 25% recurrence rate after 6 months of follow‐up (*p* < 0.01).

In one study, PUVA therapy showed comparable efficacy to intralesional corticosteroids. It was advised that a maximum of three treatment sessions would be optimal, with an interval of 3 months, or at least 16 sessions if conducted biweekly (*p* = 0.01).

The erbium‐glass laser demonstrated significant increases in hair diameter and density in AGA, both as monotherapy and in combination therapy with PRP or topical minoxidil. The intervention should last a minimum of 4 months for optimal results (*p* < 0.01). One study noted that no significant increase in hair diameter was observed, attributed to a shorter treatment period of 10 weeks compared to other studies (*p* > 0.05).

Thulium laser treatment, whether as monotherapy or in combination, also resulted in significant increases in hair thickness and density, although these effects showed a gradual decrease after completing treatment, albeit not returning to baseline during the maximum 12‐week follow‐up (*p* < 0.05).

LLLT for AGA was effective in enhancing hair density and diameter, whether as monotherapy or adjunctively. In a study on female pattern hair loss (FPHL), LLLT significantly reduced scalp oil secretion compared to minoxidil (*p* < 0.01). While three studies indicated a probable synergistic effect of LLLT combined with minoxidil, one study comparing the two therapies for male pattern hair loss found no statistically significant differences (*p* > 0.05).

LLLT effects on lichen planopilaris were evaluated in three studies, resulting in significant reductions in the lichen planopilaris activity index (LPPAI), alongside increases in terminal hair thickness and the number of thick hairs (*p* < 0.01). Treatment of frontal fibrosing alopecia with superluminescent diodes led to significant reductions in the frontal fibrosing alopecia severity score (FFASS) (*p* < 0.01), along with improvements in hyperkeratosis, pain, itching, and burning sensation, although perifollicular erythema showed no significant change (*p* > 0.05). However, LLLT did not yield positive results in treating telogen effluvium, with no significant change observed in hair count from baseline (*p* > 0.05).

### 3.4 Side Effects

The most common side effects of LLLT reported in the studies included a warm sensation at the site of treatment and scalp tenderness. Other complications, such as dry hair or skin during treatment, pruritus, stinging sensations, headaches, and slight darkening of hair (notably with blue LED), were also observed. PUVA therapy was associated with side effects like pain, hyperpigmentation, itching, blistering, and erythema. Mild erythema was commonly seen in laser or light therapies, including NBUVB, erbium‐glass laser, thulium laser, and 308‐nm excimer laser treatments. Additional complications included transient itching, desquamation, dandruff, and burning sensations.

## 4 Discussion

In this systematic review, we aimed to collect the existing information about alopecia treatment using all kinds of laser or light devices, comparing their results and probable side effects. In 2020 Roohaninasab et. al. conducted a systematic review of the available data about PRP efficacy on different types of alopecia [76]. But to our knowledge, this systematic review is the only one, which has reviewed existing literature about all types of laser/light therapy for all types of alopecia altogether. We categorized existing data into four subgroups alopecia areata (AA), androgenic alopecia (AGA), cicatricial alopecia, and telogen effluvium (TE).

In the AA subgroup, 4 of 26 studies were about narrow‐band UVB, five about topical psoralen plus UVA, and five about 308‐nm excimer laser. Based on the literature, among light/laser sources for AA excimer laser has the most prominent effect as it can be used as monotherapy or as an alternative treatment for more severe and resistant AA cases like alopecia totalis and universalis. In two retrospective studies 308‐nm excimer laser was used in alopecia universalis and other severe forms of AA resistant to other treatments, which resulted in an average 40% efficacy with 25% recurrence in 6 months follow up. On average excimer laser was used twice a week for 12 weeks with 4%–44% efficacy without considerable side effects except mild pain, erythema, desquamation, pruritus, and hyperpigmentation. During the average 4‐month follow up no relapse or recurrence was seen, but some patients indicated progression of the disease by exhibiting new patches elsewhere. In one study excimer laser was administered as an adjuvant therapy with topical minoxidil solution. Among four studies about the efficacy of NBUVB on AA, there are two randomized control trials, one retrospective, and one interventional study. NBUVB is generally used in patchy AA, which is resistant to topical treatments as an adjuvant or alternative therapy. The synergistic effect of NBUVB with topical calcipotriol and intralesional triamcinolone was also evaluated. On average NBUVB was administered twice a week for 12 weeks with 20%–53% efficacy without significant side effects except pruritus or localized erythema. Only one of these four studies had a follow‐up period of 6 weeks, in which no recurrence was observed and response and pigmentation were increased PUVA is also indicated in severe or recalcitrant types of AA as the other types of light/laser therapy in AA. In three retrospectives, one RCT, and one interventional study in this area, on average PUVA was administered twice a week for 16 weeks with an efficacy of 25%–75% without significant side effects except for scalp pain and tenderness, hyperpigmentation, and erythema. In one study oral cyclosporine was added to PUVA therapy, which led to more side effects such as gastrointestinal disturbances. Although the efficacy was not more than simple PUVA therapy in other studies. After an average of 3 months follow‐up no recurrence was observed, but after 1 year follow‐up about 20% of patients had relapse. In two prospective interventional studies light therapy (low level and polarized) was used three times a week for 4–12 weeks, which was effective in increasing hair counts with no significant side effects, and after 8 weeks of follow‐up, no recurrence was observed. No recurrence after light therapy in pediatric AA was also reported in the systematic review conducted by Behrangi et al. [77] after 6 months. The most common modality used in the AGA subgroup was low‐level light/laser therapy, which has a probable synergistic effect with topical minoxidil solution. However, its usage as monotherapy has also significant outcomes. The same positive results are reported by Afifi et. al., in their systematic review of the LLLT effect on AGA [16]. In the second place after LLLT, erbium glass laser, when used as AGA treatment, also has a significant positive effect in increasing hair count and diameter. Generally, light therapy is used as an alternative or additional therapy besides topical minoxidil solution in patients with no history of antiandrogen drugs or hair transplantation. In 17 studies about the LLLT effect on AGA, there are 10 RCTs, one retrospective, and one pilot study and eight studies are conducted on only male patients and six studies on female ones. The average severity of AGA was average Norwood‐Hamilton III–V for males and I–III Savin–Ludwig for females. The average administration of LLLT was every other day for 24 weeks with an average efficacy of 50% without significant side effects except an initial increase in hair shedding, headache, mild scalp tenderness, slight darkening of hair, erythema, acne, eczema, pruritus, and warm sensation. After an average of 3 months of follow up no recurrence was observed in patients. In six erbium: glass laser studies, there are one RCT, one retrospective, one pilot, and three prospective trials. understudied cases in two studies were only males and in one study only female patients were included. One study used 1540 nm and the other used 1550 nm Erbium: glass laser. This laser modality was used concomitantly with topical minoxidil solution in male patients or with PRP every other session. ER: glass laser was administered on average every 2 weeks for 20–24 weeks with efficacy in increasing hair density more than hair diameter, without considerable side effects except mild erythema, mild erosion, pruritus, burning sensation, and dandruff. In one study after 4 months of follow‐up, hair density decreased to baseline levels but in another study after 3, 6, and 12 months of follow‐up, the duration of positive effects was ongoing.

Available data on cicatricial alopecia and telogen effluvium is scarce which makes a generalized interpretation difficult. However, LLLT seems to be effective in LPPAI and FFAS reduction. Laser and light therapy is used as additive therapy in most articles on cicatricial alopecia. In five studies about cicatricial alopecia, one study was about ALA‐PDT, three about LLLT, and one was about CO_2_ and erbium glass laser. In three LLLT studies, there was one case series, one pilot study, and one prospective interventional study. Daily administration of LLLT for at least 6 months in LPP patients had significant efficacy in reducing LPPAI score without considerable side effects except for warmth and stinging sensation and dry skin. No follow‐up observation was reported, but the positive results didn't show a reduction until the end of the intervention. In the only prospective interventional study about TE, LLLT was administered twice a week for 16 weeks in female patients with acute or chronic TE, which had 70% efficacy according to patients but no significant efficacy with minor side effects such as heat sensation, scalp tenderness, headache and dryness of hair through treatment duration. In 28 weeks of follow‐up hair count decreased from the end of the treatment period. The current study had some limitations that include, there was significant heterogeneity between included studies, Therefore, it was not possible to determine a pooled estimation between the studies, Also The available data in the TE category is less than expected, so it can affect the conclusion. Another limitation was the design of included studies. Most of the studies had a small sample size and did not perform randomization So more future studies with randomized clinical trial designs with a larger sample size should be conducted about laser/light therapy in Alopecia especially in telogen effluvium and different types of cicatricial alopecia fields.

## 5 Conclusion

Although care must be taken in interpreting data, the majority of studies showed good outcomes in hair diameter and density increase and patient satisfaction. Light/laser therapy can be useful in recalcitrant and severe types of AA with an average efficacy of about 50% and with highest efficacy of 75%. Also, in female and male pattern AGA, light therapy especially LLLT has an efficacy of about 50% as an alternative therapy, which shows synergistic effects in combination with topical minoxidil solution. LLLT and other types of light therapy can be also used as adjuvant therapy in cicatricial alopecia patients. However, this treatment is not efficient for TE according to available data. There is a need for further research to evaluate the efficiency of light/laser therapy in telogen effluvium and cicatricial alopecia. In conclusion, laser/light therapy is assumed as an alternative treatment for different types of alopecia, especially when patients are not willing to use medical or surgical options. Light/laser therapy is also safe and the critical adverse effects of these treatment methods have not been reported. More research is needed to find the exact features of patients, which are ideal candidates for such treatment.

## Author Contributions

**Mohammad Amin Jafari:** conceptualization, writing–review and editing, data curation, methodology, validation, investigation, writing–original draft. **Ghazal Bazgir:** writing–review and editing, data curation, writing–original draft. **Fatemeh Sadat Hosseini‐Baharanchi:** formal analysis, data curation, writing–original draft. **Alireza Jafarzadeh:** writing–review and editing. **Azadeh Goodarzi:** conceptualization, investigation, writing–original draft, writing–review and editing, data curation, supervision, formal analysis, project administration, methodology.

## Conflicts of Interest

The authors declare no conflicts of interest.

## Transparency Statement

The lead author Azadeh Goodarzi affirms that this manuscript is an honest, accurate, and transparent account of the study being reported; that no important aspects of the study have been omitted; and that any discrepancies from the study as planned (and, if relevant, registered) have been explained.

## Data Availability

The data that support the findings of this study are available from the corresponding author upon reasonable request.
